# A Bar to Malpractice Suits

**Published:** 1884-08

**Authors:** 


A BAR TO MALPRACTICE SUITS.—A case tried not long since in one of the Western States brought up the question whether a successful suit by a physician for the value of his services did not bar any subsequent suit by the patient for malpractice, and it was held that it did. The first suit was litigated to decide the point of the value of the services, and although the charge of malpractice was not directly made as in the second suit, yet if there had been any malpractice there would, of course, have been no value to the services. The failure to make the charge in the first suit was held to forever prevent its being raised afterwards.—*Med. and Surg. Reporter.*

